# A rapid and scalable method for selecting recombinant mouse monoclonal antibodies

**DOI:** 10.1186/1741-7007-8-76

**Published:** 2010-06-04

**Authors:** Cécile Crosnier, Nicole Staudt, Gavin J Wright

**Affiliations:** 1 Cell Surface Signalling Laboratory, Wellcome Trust Sanger Institute, Cambridge CB10 1HH, UK

## Abstract

**Background:**

Monoclonal antibodies with high affinity and selectivity that work on wholemount fixed tissues are valuable reagents to the cell and developmental biologist, and yet isolating them remains a long and unpredictable process. Here we report a rapid and scalable method to select and express recombinant mouse monoclonal antibodies that are essentially equivalent to those secreted by parental IgG-isotype hybridomas.

**Results:**

Increased throughput was achieved by immunizing mice with pools of antigens and cloning - from small numbers of hybridoma cells - the functionally rearranged light and heavy chains into a single expression plasmid. By immunizing with the ectodomains of zebrafish cell surface receptor proteins expressed in mammalian cells and screening for formalin-resistant epitopes, we selected antibodies that gave expected staining patterns on wholemount fixed zebrafish embryos.

**Conclusions:**

This method can be used to quickly select several high quality monoclonal antibodies from a single immunized mouse and facilitates their distribution using plasmids.

## Background

The ability of antibodies to bind, with high selectivity and affinity, to diverse chemical structures has made them an extremely important tool for biomedical research [1]. Their value is underlined by the numerous ongoing international efforts, both academic and commercial, to produce large antibody resources for the scientific community [2]. A great deal of effort has, therefore, been applied to both improve antibody selection and develop other types of affinity binders including the use of other protein scaffolds [3-5] or nonproteinaceous reagents [6].

Ideally, a binding reagent should have a high affinity and specificity for its target and be produced quickly with little resource. However, these two parameters are usually traded against each other. Monoclonal antibodies raised *in vivo *within the mammalian immune system are matured by somatic hypermutation and, therefore, usually produce high affinity antibodies, but they are relatively slow to isolate. Conversely, antibodies selected from *in vitro *libraries can be produced more rapidly but are typically of lower binding strength due to the lack of affinity maturation. Importantly, antibodies that have low affinities are often unsuitable for applications commonly used by cell and developmental biologists, such as wholemount immunohistochemistry, because of the extensive washing steps required by these protocols.

Although polyclonal antisera are an efficient and rapid way of raising high affinity antibodies, they are finite and can suffer from substantial batch-to-batch variation [7]. By providing essentially limitless amounts of a defined reagent, monoclonal antibodies selected from an immunized animal therefore remain the reagent of choice. They are, however, generally regarded as unsuitable for building large resources because of the requirement for long immunization schedules and a large tissue culture demand. Despite efforts to alleviate some aspects of this procedure, the large tissue culture burden of subsequent hybridoma cell cloning remains a significant barrier for large scale production [8,9]. Also, the use of chemically synthesized peptides as antigens is rapid and cost effective but, in many cases, these reagents do not adequately mimic the shape of a natively folded protein. Antibodies raised against peptides, therefore, often recognize only denatured proteins (for example on western blots) and do not stain native proteins found in wholemount fixed tissue, thus limiting their usefulness.

The zebrafish is an increasingly popular model organism used to understand early vertebrate developmental processes and model diseases [10-12]. The amenability to forward genetics and the ability to produce large numbers of translucent embryos that rapidly develop externally has enabled the genetic dissection of most vertebrate organ systems [13,14]. While significant advances have been made in genetic methods [15,16], the paucity of high quality antibodies is considered a significant limitation for zebrafish research [17]. The few antibody reagents available to zebrafish researchers have mostly come from 'shotgun' approaches where zebrafish tissue lysates were used as immunogens [9,18,19]. While this approach is valuable, the target antigen is not immediately known and the isolated antibodies frequently recognize highly immunogenic fish-specific glycans, which may not be protein specific [18,20].

In order to identify novel signalling pathways initiated by extracellular protein interactions between cell surface and secreted receptor-ligand pairs, we have recently compiled a library containing the ectodomain fragments of 249 zebrafish glycoproteins which mainly belong to the immunoglobulin (IgSF) and leucine-rich repeat (LRR) families [21,22]. This protein library represents a useful source of antigens to raise antibodies that can be used not only as differentiation markers but also to isolate intact living cells from suspensions, or ectopically activate signalling pathways. Furthermore, expressing these entire ectodomains in mammalian cells ensures that structurally important posttranslational modifications, such as glycosylation and disulfide bonds, are added and the proteins are therefore more likely to be natively folded.

Here, we report the development and implementation of a convenient and scalable method for selecting mouse monoclonal antibodies against several zebrafish cell surface receptor proteins in parallel. We have developed a procedure for the rapid cloning of both the functionally rearranged heavy and light chains into a single expression plasmid from a small number of hybridoma cells. By screening for formalin-resistant epitopes we show that this procedure produces antibodies that display the expected staining on wholemount fixed tissues. The use of a single antibody expression plasmid facilitates the distribution of these reagents making it suitable for compiling an antibody resource.

## Methods

### Protein production and purification

All zebrafish receptor ectodomain fragments were produced in HEK293E cells as rat Cd4 domains three- and four-tagged secreted proteins. The Cd4 tag was followed by either a six histidine-tag for purification or an enzymatic biotinylation site and cotransfected with the BirA enzyme, essentially as described [21]. Supernatants were harvested 6 days after transfection, filtered and stored at 4°C. Histidine-tagged proteins were purified with HisTrap HP columns (GE Healthcare, Buckinghamshire, UK) as previously described [21]. Proteins were over 90% pure as determined by SDS-PAGE and quantified by measuring absorbance at 280 nm. Antibodies were isotyped using the ISO-2 isotyping kit (Sigma, MO, USA).

### Immunizations

Six-week-old male BALB/c mice were immunized subcutaneously with pools of five purified His-tagged proteins (5 μg each protein per immunization) in complete Freund's adjuvant (once) and incomplete adjuvant (three times). Mice selected for hybridoma production were boosted intraperitoneally 3 days before dissecting the spleen.

### Cell culture and hybridoma generation

The SP2/0 myeloma and SP2/mIL6 cell lines were grown in advanced DMEM/F12 medium (Invitrogen, CA, USA) supplemented with 20% fetal bovine serum, penicillin (100 U/mL), streptomycin (100 μg/mL) and L-glutamine (2 mM). SP2/mIL6 conditioned medium was harvested every 3 days. Following spleen dissection and dissociation, 10^8 ^splenocytes were fused to 10^7 ^SP2/0 myeloma in 50% PEG (PEG 1500, Roche, Hertfordshire, UK), using standard procedures. The resulting hybridomas were plated over 10 96-well plates and initially grown in advanced DMEM/F12 medium (Invitrogen) supplemented with 20% fetal bovine serum, 20% Sp2/mIL6 conditioned medium, penicillin (100 U/mL), streptomycin (100 μg/mL) and L-glutamine (2 mM) before addition of hypoxanthine aminopterin thymidine (HAT) selection medium 24 h after the fusion; after a regular exchange of selection medium for 2 weeks, hybridoma supernatants were harvested for screening. Positive clones were re-plated and grown for a further 5 days.

### Antibody screening

Hybridoma supernatants were screened using an ELISA-based assay. Briefly, the biotinylated ectodomains of the injected proteins were bound to streptavidin-coated plates (NUNC, NY, USA) and incubated for 1 h with 50 μL hybridoma supernatant diluted 1:2 in phosphate buffered saline (PBS) and 0.2% bovine serum albumin (BSA). The plates were washed in PBS/0.1% Tween20 (PBST) before incubation with an anti-mouse immunoglobulin antibody coupled to alkaline phosphatase (Sigma) for 1 h at room temperature. After washes in PBST and PBS, positive wells were detected with *p*-nitrophenyl phosphate at 1 mg/mL. In order to test for fixation-sensitivity, protein ectodomains were incubated between 1 min and 2 h in 4% formalin before the addition of the hybridoma supernatant.

### Plasmid construction

The immunoglobulin expression vector was constructed from a derivative of pTT3 [23] that contains an expanded multiple cloning site. The leader sequence of the mouse variable κ light chain 7-33 was introduced by annealing of primers 1 and 2 (Table 1) and cloning between the *PmeI *and *NotI *restriction sites for production of the recombinant antibody light chain. The constant region of the mouse immunoglobulin G_1 _(IgG_1_) isotype was amplified from mouse genomic DNA using primers 49 and 50, and inserted between the *AscI *and *XbaI *restriction sites for expression of the recombinant heavy chain.

**Table 1:** Primers used for the generation of recombinant mouse monoclonal antibodies.

Primers used for the generation of a leader sequence for the κ light chains	
1	GCCACCATGGAGTTTCAGACCCAGGTACTCATGTCCCTGCTGCTCTGCATGTCTGGTGC
2	GGCCGCACCAGACATGCAGAGCAGCAGGGACATGAGTACCTGGGTCTGAAACTCCATGGTGGC
**Degenerate forward primers used for the amplification of rearranged κ light chains**	
3	TCAATAGTTGAACATAGCGGCCGCASAAAWTGTKCTCACCCAGTC
4	TCAATAGTTGAACATAGCGGCCGCAGAWATTGTGCTMACTCAGTC
5	TCAATAGTTGAACATAGCGGCCGCAGACATTGTGCTRACACAGTC
6	TCAATAGTTGAACATAGCGGCCGCAGACATTGTGATGACMCAGTC
7	TCAATAGTTGAACATAGCGGCCGCAGAYATCMAGATRAMCCAGTC
8	TCAATAGTTGAACATAGCGGCCGCAGAYATCCAGATGAYTCAGTC
9	TCAATAGTTGAACATAGCGGCCGCAGATATCCAGATGACACAGAC
10	TCAATAGTTGAACATAGCGGCCGCAGACATTGTGCTGACCCAATC
11	TCAATAGTTGAACATAGCGGCCGCAGACATYSTRATGACCCARTC
12	TCAATAGTTGAACATAGCGGCCGCAGATRTTKTGATGACYCARAC
13	TCAATAGTTGAACATAGCGGCCGCAGAYATTGTGATGACBCAGKC
14	TCAATAGTTGAACATAGCGGCCGCAGATATTGTGATAACCCAGGA
15	TCAATAGTTGAACATAGCGGCCGCAGACATCYTGCTGACYCAGTC
16	TCAATAGTTGAACATAGCGGCCGCAGAAAWTGTGYTGACCCAGTC
17	TCAATAGTTGAACATAGCGGCCGCAGAAACAACTGTGACCCAGTC
18	TCAATAGTTGAACATAGCGGCCGCAGACATTRTGATGWCACAGTC
19	TCAATAGTTGAACATAGCGGCCGCAGACATCCAGMTGACMCARTC
**Reverse primer used for the amplification of rearranged κ light chains**	
20	GGATACAGTTGGTGCAGCATCAGCCC
**Primers used for the generation of the joining fragment**	
21	GGGCTGATGCTGCACCAACTGTA
22	TCAAGTGCAAAGACTCACTTTATTG
23	CAATAAAGTGAGTCTTTGCACTTGATAGTTATTAATAGTAATCAATTACG
24	TAGCAGAACAGGCAGCTTCATGGTGGCCTGGGAGTGGACACCTGTGGAGAG
25	ATGAAGCTGCCTGTTCTGCTAGTG
26	ACTGCTTGAGGCTGGACTCGTGAACAATAGCAGC
**Degenerate forward primers used for the amplification of rearranged heavy chains**	
27	TTCACGAGTCCAGCCTCAAGCAGTGAKRTRCAGCTTMAGGAGTC
28	TTCACGAGTCCAGCCTCAAGCAGTGAGKTYCAGCTBCAGCAGTC
29	TTCACGAGTCCAGCCTCAAGCAGTCAGGTGCAGMTGAAGSAGTC
30	TTCACGAGTCCAGCCTCAAGCAGTGAGRTCCAGCTGCAACARTC
31	TTCACGAGTCCAGCCTCAAGCAGTCAGGTYVAGCTGCAGCAGTC
32	TTCACGAGTCCAGCCTCAAGCAGTCAGGTYCARCTGCAGCAGTC
33	TTCACGAGTCCAGCCTCAAGCAGTGAGGTGMAGCTGGTGGAATC
34	TTCACGAGTCCAGCCTCAAGCAGTGAVGTGMWGCTSGTGGAGTC
35	TTCACGAGTCCAGCCTCAAGCAGTGARGTGCAGCTGKTGGAGWC
36	TTCACGAGTCCAGCCTCAAGCAGTGAGGTGAAGCTGATGGAATC
37	TTCACGAGTCCAGCCTCAAGCAGTGAGGTGCAGCTTGTTGAGTC
38	TTCACGAGTCCAGCCTCAAGCAGTGAGGTGAAGCTTCTCRAGTC
39	TTCACGAGTCCAGCCTCAAGCAGTGAAGTGAARMTTGAGGAGTC
40	TTCACGAGTCCAGCCTCAAGCAGTCAGGTTACTCWGAAAGWGTCTG
41	TTCACGAGTCCAGCCTCAAGCAGTCAGGTCCAACTGCAGCAGCC
42	TTCACGAGTCCAGCCTCAAGCAGTGATGTGAACCTGGAAGTGTC
43	TTCACGAGTCCAGCCTCAAGCAGTCAGATCCAGTTSGTRCAGTC
44	TTCACGAGTCCAGCCTCAAGCAGTGAGGTRCAGCTKGTAGAGAC
**Reverse primers used for the amplification of rearranged heavy chains**	
45	CTATTCTAGCTAATCTAGGCGCGCCGAGGAGACGGTGACCGTGGTCC
46	CTATTCTAGCTAATCTAGGCGCGCCGAGGAGACTGTGAGAGTGGTGC
47	CTATTCTAGCTAATCTAGGCGCGCCGCAGAGACAGTGACCAGAGTCC
48	CTATTCTAGCTAATCTAGGCGCGCCGAGGAGACGGTGACTGAGGTTC
**Primers used for the amplification of the IgG1 constant region**	
49	ATATCACGGCGCGCCCGACACCCCCATCTGTCTATCCA
50	GACTGATCTAGATCATTTACCAGGAGAGTGGGAGAG
**Primers used for fusion polymerase chain reaction**	
51	TCAATAGTTGAACATAGCGGCCGC
52	CTATTCTAGCTAATCTAGGCGCGCCG
**Primer specific for the aberrant κ light chain from SP2/0 myeloma**	
53	GGATGCTGCAACCTATTACTGTCAGCACATTAGGGAGCTTACACG

The primers used to amplify the rearranged heavy and kappa light variable regions were designed using recent genome sequence of the mouse immunoglobulin regions: NG_005612 for the κ light locus and NG_005838 for the heavy locus. Degenerate base code: M = A+C; R = A+G; W = A+T; S = C+G; Y = C+T; K = G+T; V = A+C+G; B = C+G+T.

### RNA preparation and reverse transcriptase polymerase chain reaction (RT-PCR)

Total RNAs were prepared from selected hybridoma using the RNAqueous micro kit (Ambion, Texas, USA) and the reverse transcription performed with a polydT primer, using SuperscriptIII (Invitrogen). The functionally rearranged light variable region of a selected hybridoma was amplified without its signal peptide sequence using a set of 17 degenerate forward primers containing a *NotI *site (primers 3 to 19) and one unique reverse primer corresponding to the constant region of the κ light chain (primer 20). Similarly, the functionally rearranged heavy variable region was amplified without its signal peptide sequence using a set of 18 degenerate forward primers (primers 27 to 44) and four reverse primers containing an *AscI *site (primers 45 to 48). Both heavy and light variable regions were amplified using Advantage2 polymerase (Clontech, CA, USA) using the PCR conditions: 94°C for 5 min; five cycles at 94°C for 45 s, 65°C for 30 s, 72°C for 1 min; 19 cycles at 94°C for 45 s, 64.5°C to 55.5°C for 30 s with 0.5°C decrement per cycle, 72°C for 1 min; and 10 cycles at 94°C for 45 s, 55°C for 30 s, 72°C for 1 min.

### Assembly of the expression vector

In order to assemble the light and heavy variable regions, a joining fragment composed of the constant region of the κ light chain and its 3' untranslated region, a cytomegalovirus (CMV) promoter and the leader sequence of the mouse variable κ light chain 1-99 was created by fusion PCR with primers 21 and 26. The κ constant region and Vκ 1-99 were amplified from mouse genomic DNA using primers 21 and 22, and 25 and 26, respectively, while the CMV promoter was amplified from the pTT3 vector with primers 23 and 24. The light and heavy variable RT-PCR products and the joining fragment were gel purified and assembled into a contiguous product by fusion PCR using KOD polymerase (Novagen, Nottingham, UK) and primers 51 and 52. PCR conditions were: 94°C for 5 min; five cycles at 94°C for 45 s, 65°C for 30 s, 72°C for 3 min; 19 cycles at 94°C for 45 s, 64.5°C to 55.5°C for 30 s with 0.5°C decrement per cycle, 72°C for 3 min; 10 cycles at 94°C for 45 s, 55°C for 30 s, 72°C for 3 min. The fusion PCR product was subsequently cloned into the immunoglobulin expression vector between the *NotI *and *AscI *restriction sites.

### Colony PCR

Plasmids containing a functional variable light and heavy chain were selected by colony PCR using primers 23, 50 and 53. Primer 53 corresponds to the aberrantly-rearranged light chain present in SP2/0 myeloma and was used to identify plasmids containing the aberrant SP2/0 light chain with a PCR product size ~2.5 kb. Plasmids containing both functional light and heavy variable fragments produced a product around 3.1 kb.

### Western blotting

Tissue culture medium, transfection medium containing the recombinant 5F11 antibody or tissue culture medium spiked with purified 5F11 from the parental hybridoma were resolved by SDS-PAGE under both reducing and non-reducing conditions before blotting onto Hybond-P PVDF membrane (GE Healthcare) overnight at 30 V. Membranes were blocked with 2% BSA in PBST and incubated with 1 μg/mL of an anti-mouse IgG-HRP conjugate (Molecular Probes, Oregon, USA) diluted in 0.2% BSA and detected with the Supersignal West pico chemiluminescent substrate (Pierce).

### Biophysical analysis of antibody affinities

Antibody dissociation rates were determined using a BIAcore T100 biosensor. Biotinylated Cd4d3 + 4-tagged recombinant proteins were immobilized onto streptavidin-coated SA sensor chip surfaces at low levels (Flrt3 ~750 RU, Jamc.2 ~400 RU, Lrrn1 ~1320 RU) and a molar equivalent of Cd4d3 + 4bio used as a reference. Purified Unc5bCd4d3 + 4-6H protein was covalently immobilized (~360 RU) to a CM5 sensor chip using the amine coupling kit (BIAcore, Buckinghamshire, UK). Dissociation curves were exported and analysed in Microsoft Excel.

### Wholemount *in situ *hybridization and immunohistochemistry

Zebrafish were maintained on a 14/10 h light/dark cycle at 28.5 °C according to the UK Home Office and local institutional regulations and staged according to Kimmel [24]. *In situ *hybridization was performed according to standard procedures [25] using 100 ng of either *unc5b*- or *flrt3*-RNA probes prepared as described [21]. Bright field images were captured on an Imager M1 microscope (Zeiss, Hertfordshire, UK). Purified parental or crude recombinant 5F11 antibody was diluted 1:500 or 1:2, respectively, in 10% goat serum, 2% BSA, 0.1% Triton X-100 in PBS and incubated with cryosections of 3-day-old formalin-fixed zebrafish embryos. For wholemount staining, zebrafish embryos were fixed overnight at 4°C in a 4% formalin solution and washed overnight in PBS/1% Triton X-100. Purified recombinant anti-Unc5b and anti-Flrt3 antibodies (0.5 mg/mL in PBS) were incubated with fixed embryos at 4°C overnight followed by three 15 min wash steps in PBST. For both cryosection and wholemount staining, an anti-mouse-Alexa568 secondary antibody was used (1:1000, Invitrogen) and tissues were counterstained with DAPI. Images of dissected and flat-mounted embryos were taken on a DM6000B confocal microscope (Leica, Wetzlar, Germany) and processed using Adobe Photoshop CS4.

## Results

### A method for the rapid selection of recombinant mouse monoclonal antibodies

**Figure 1:**
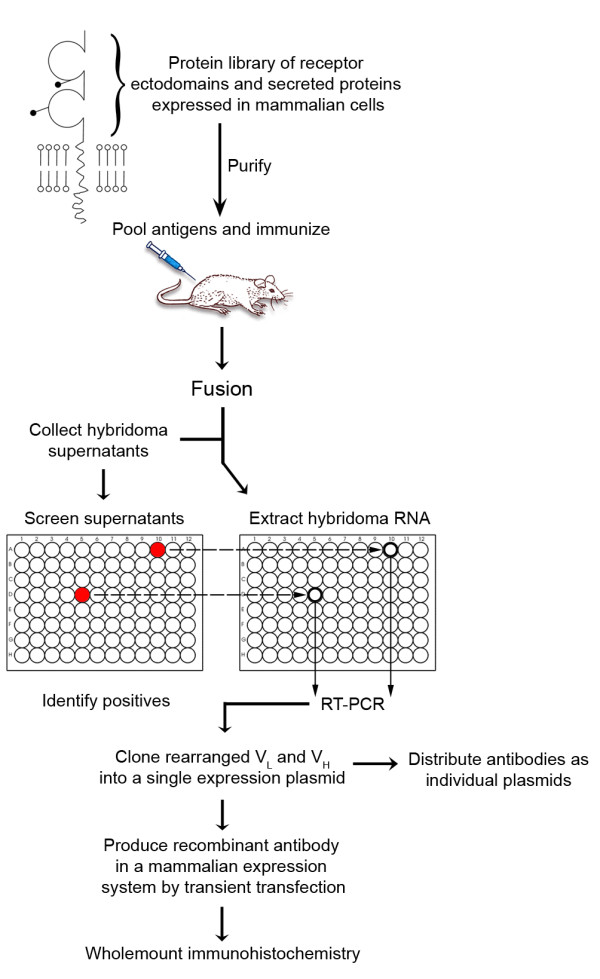
**Overview of a scalable procedure to select recombinant mouse monoclonal antibodies**. Purified ectodomain fragments of zebrafish cell surface and secreted proteins expressed in mammalian cells were normalized, pooled and used to immunize mice. Hybridomas were generated by cell fusion, and supernatants screened for positives using ELISA. The rearranged light and heavy antibody chains were amplified from RNA extracted from small numbers of hybridoma cells by reverse transcription-polymerase chain reaction and cloned into a single expression plasmid. The recombinant monoclonal antibodies were produced by transfecting mammalian cells before testing for fixation sensitivity and wholemount staining.

Our aim was to rapidly select monoclonal antibodies of high affinity and specificity that could be used in wholemount immunohistochemistry protocols and could be easily distributed. The successful procedure is outlined in Figure 1 and is based around standard animal immunization and hybridoma technology, which enabled the selection of antibodies of a given specificity and ensured that they were affinity matured. Throughput was increased by immunizing mice with pools of antigens from our library of zebrafish cell surface and secreted proteins, so that antibodies to several proteins could be selected simultaneously from a single animal. In order to circumvent the burdensome tissue culture cloning procedures usually associated with the isolation of hybridomas, the productively rearranged heavy and light chain loci were amplified from small numbers of selected hybridoma cells by RT-PCR. The amplified light and heavy chains were then joined into a contiguous PCR product and cloned into a mammalian expression plasmid so that large amounts of antibody could be quickly produced by transiently transfecting mammalian cells. Recombinant antibodies were then validated by staining fixed wholemount zebrafish embryos and the patterns checked for congruence with the known *in situ *expression profiles.

### Proof-of-principle: cloning of the 5F11 monoclonal antibody

In order to clone productively rearranged antibody loci from hybridomas, we expanded a set of primers to mouse immunoglobulin V-regions originally published by Krebber [26] by using more recent genomic sequence information (Table 1). This new set of primers can recognize 97 and 98% of functional heavy and kappa (κ) light chain V-regions, respectively. Lambda light chains only constitute 5% of those used in mouse immunoglobulins [27] and primers were, therefore, not designed to this light chain. Hybridomas were generated using the SP2/0 myeloma cell line, which lacks transcription from the heavy chain locus due to a genomic inversion [28], but is known to transcribe an aberrantly rearranged κ light chain [29]. We anticipated that this aberrant light chain as well as the possible transcription of non-productively rearranged heavy or light chain loci present in the original B-cell could be amplified and would have to be identified and excluded.

**Figure 2:**
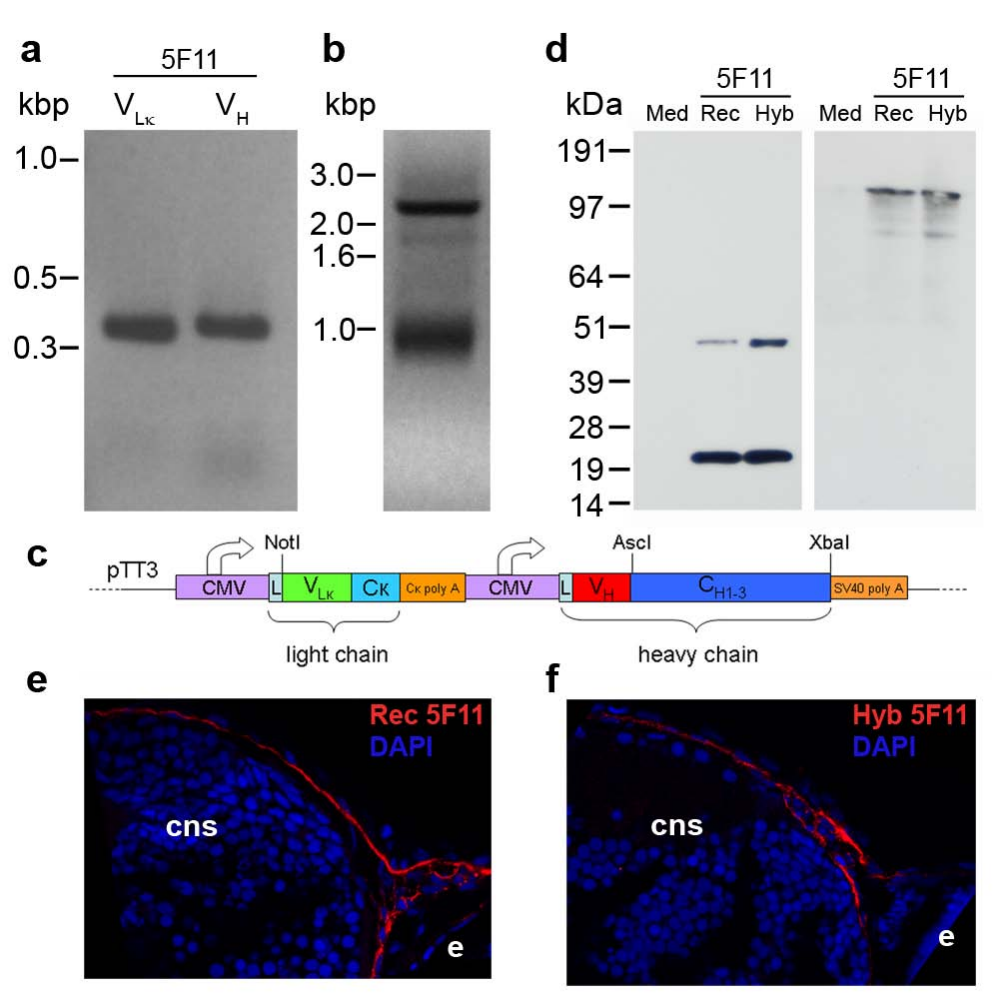
**Cloning of the 5F11 monoclonal antibody**. (a) Reverse transcriptase-polymerase chain reaction (PCR) amplification of the variable light (V_Lκ_) and heavy (V_H_) antibody chains from the 5F11 hybridoma. (b) Assembly of the 5F11 V_Lκ _and V_H _regions by fusion PCR through a joining fragment resulting in a 2.4 kbp product. (c) Schematic of the plasmid encoding the recombinant antibody with the fusion PCR product cloned between *NotI *and *AscI*. (d) Western blot run under reducing (left panel) or non-reducing (right panel) conditions: tissue culture medium (Med), supernatant of cells producing the recombinant 5F11 antibody (Rec) or tissue culture medium spiked with the 5F11 monoclonal antibody purified from the parent hybridoma (Hyb). Staining of the recombinant 5F11 [Rec 5F11, (e)] is indistinguishable from that of the parent hybridoma [Hyb 5F11, (f)], red. Nuclei are counterstained in blue (DAPI). Abbreviations: cns = central nervous system; e = eye; CMV = cytomegalovirus promoter; L = leader sequence; C = constant region; V = variable region.

As a proof of principle, we used a hybridoma (5F11) which secretes a mouse IgG_1 _recognizing an epitope restricted to zebrafish basement membranes [18]. Using our set of primers, we successfully amplified PCR products of expected sizes corresponding to the productively rearranged variable heavy and light chain regions (Figure 2a). These two products were then assembled by fusion PCR using a joining fragment that contained (5' to 3') a κ light chain constant region and its polyadenylation signal, a CMV promoter and a signal peptide for the heavy chain (Figure 2b). The primers used for the fusion PCR contained the rare-cutting restriction enzymes *NotI *and *AscI *to facilitate cloning into a single mammalian expression plasmid, which included a signal peptide for the light chain and the constant region of the mouse IgG_1 _antibody isotype. The final construct contained both light and heavy chains, each flanked by a CMV promoter and polyadenylation sequence (Figure 2c). Although some clones contained the aberrant SP2/0-derived light chain, we selected a clone containing both productively rearranged light and heavy chains.

Western blotting of supernatants from transiently transfected HEK293E cells under reducing and non-reducing conditions showed that the heavy and light chains were stoichiometrically balanced in their expression level and capable of association (Figure 2d). The unpurified tissue culture supernatant was used in place of the primary antibody in an immunohistochemistry protocol using cryosections of 3-day old zebrafish larvae and strong staining of the basement membranes was observed with the recombinant antibody (Figure 2e) which was indistinguishable from that obtained with antibodies secreted by the original hybridoma (Figure 2f). These data validate the overall strategy for producing recombinant antibodies from a single expression plasmid.

### Multiple different antibodies per fusion using pooled antigen immunization

To increase the throughput of monoclonal antibody generation and show that antibodies could be cloned from hybridomas at an early stage after selection, we immunized mice with two pools of five different proteins selected from our library, and performed the fusions using standard procedures (Table 2). Hybridoma supernatants were screened by ELISA using the appropriate biotinylated recombinant proteins immobilized on streptavidin-coated 96-well plates. Hybridomas secreting antibodies of interest were expanded for 5 days before removing ~ 10^5 ^cells for total RNA extraction; the supernatant was retained for antibody isotyping and future comparisons with recombinant antibodies. For each hybridoma, cDNA was synthesized and used to independently amplify both light and heavy chains before joining them in a single expression construct. All six hybridomas for which a heavy chain was cloned contained a productively rearranged V_H _region. Only one also had an aberrant heavy chain transcript, which presumably originated from an unproductive rearrangement of the other heavy chain allele within the fusing B-cell. The only aberrant κ light chain identified originated from SP2/0 and was present in 26% to 70% of plasmids depending on the hybridoma studied; these were quickly identified and eliminated by colony PCR using a primer specific to this sequence. In general, cloning of the productively rearranged antibody chains was readily achieved (Table 2). In two cases (clones 1.2.3 and 1.4.2) cloning was halted due to the successful completion of a different antibody for that antigen. In the three other cases where antibody cloning was not successful, two were due to very small hybridoma colonies which stopped dividing (clones 1.1.1 and 1.3.1) or because the antibody secretion was lost (clone 2.4.1).

**Table 2:** A summary of the antibodies selected in this study.

	Target	Antigen	Antibody name	Clone	V_L_	V_H_	Formalin sensitive?
Fusion 1	1.1	Jamc.1		1.1.1	V14-111+J2	ND	ND
	
	1.2	Unc5b	SI1-Unc5b	1.2.1	V17-127+J4	V2-2+D1+J4	Y
			
			SI2-Unc5b	1.2.2	V5-48+J5	V1-14+D1+J4	N
			
				1.2.3	Cloning halted		
	
	1.3	Lrrc4a		1.3.1	Clone lost		
	
	1.4	Flrt3	SI3-Flrt3	1.4.1	V3-7+J1	V1-66+D2+J3	N
			
				1.4.2	Cloning halted		
	
	1.5	Appa		No positive clone			

Fusion 2	2.1	Jamb.1		No positive clone			
	
	2.2	Jamc.1		No positive clone			
	
	2.3	Jamc.2	SI4-Jamc.2	2.3.1	V10-96+J1	V3-2+D2+J1	N
	
	2.4	Vasn		2.4.1	N.D.	V5-6+D2+J2	ND
	
	2.5	Lrrn1	SI5-Lrrn1	2.5.1	V4-57+J5	V1-77+D3+J2	N

The pooled antigens used in the immunizations are numbered, and details of the V, D and J segments identified are listed for each antibody. Antibodies were numerically named with the SI prefix for Sanger Institute.

ND = not determined

Single plasmids containing productively rearranged light and heavy chains were transfected into HEK293E cells and the supernatant was tested by ELISA to ensure that the recombinant antibodies retained specificity for their antigens. After purification from bulk cultures, yields ranged from 10 μg/mL (SI4-Jamc.2) to 40 μg/mL (SI3-Flrt3). Of the nine different immunized proteins, 10 positive hybridoma supernatants were identified for seven antigens and five recombinant antibodies that retained binding specificity were isolated.

**Figure 3:**
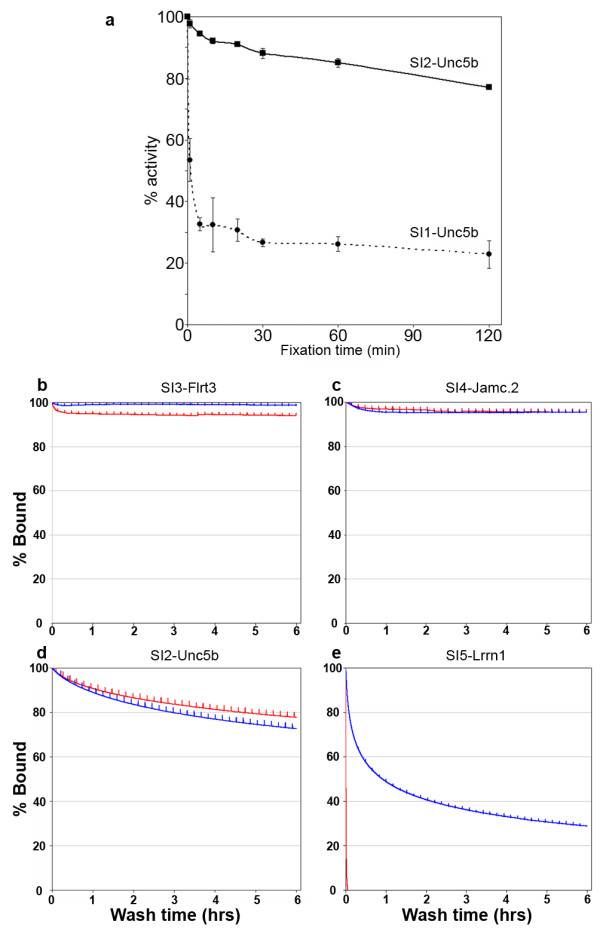
**Sensitivity to formalin treatment and affinity measurement of recombinant antibodies**. (a) Recombinant antibodies SI1-Unc5b and SI2-Unc5b were tested for formalin fixation sensitivity by ELISA following antigen treatment with a 4% formalin solution. (b-e) The dissociation rates of the recombinant (red line) antibodies were compared to the original hybridoma antibodies (blue line) using surface plasmon resonance. Antibodies are: (b) SI3-Flrt3, (c) SI4-Jamc.2, (d) SI2-Unc5b and (e) SI5-Lrrn1. In order to facilitate comparison, binding at saturation was normalized to 100% and rebinding was minimized by immobilizing low levels of target protein and washing at 37°C using high flow rates (100 μL/min).

Fixation of biological samples, usually necessary to maintain tissue architecture for subsequent analysis, can modify the chemical structure of the protein resulting in the destruction of antibody epitopes. This is a particular problem for monoclonal antibodies which recognize a single epitope. We therefore screened each antibody against recombinant proteins that had been formalin-treated for different lengths of time. Of the two antibodies that recognize the Unc5b receptor, one (SI2-Unc5b) was insensitive and the other (SI1-Unc5b) sensitive to formalin-treated proteins (Figure 3a). We found, however, that most epitopes were insensitive to formalin-fixation (Table 2).

### Recombinant and parental hybridoma IgG antibodies have comparable affinities

Antibodies cloned and produced recombinantly will differ in several respects from those secreted by hybridomas. First, all antibodies contain two amino acid changes (KT to RP) at the junction of the V_H _and C_H1 _domains due to the incorporation of the *AscI *cloning site. The degenerate primers used to amplify the V_L _and V_H _are also likely to introduce amino acid changes at the N-terminus of each chain. The antibody isotype, if not originally an IgG_1_, will be altered, and finally the antibodies produced in HEK293E cells will contain different glycans. In order to determine the consequences of these changes on the antibody affinity, we compared each recombinant antibody to the original hybridoma supernatant using a BIAcore instrument. Each antigen and a control were immobilized at approximate molar equivalence on a sensor chip; either the hydridoma or the recombinant antibody was injected until saturation had been achieved (data not shown) and the rate of dissociation of the antibody was then followed for several hours. Of the four recombinant antibodies tested, three (clones SI2-Unc5b, SI3-Flrt3, and SI4-Jamc.2) were essentially indistinguishable from the original hybridoma demonstrating that the changes due to the *AscI *site at the V_H_-C_H1 _junction, and those due to the V_L _and V_H_-region degenerate primers, do not affect antibody affinity, at least for these antibodies (Figure 3b-d). In contrast, the affinity of the recombinant SI5-Lrrn1 antibody was reduced >200-fold (Figure 3e). Isotyping revealed that the original hybridoma antibody was an IgM which would have more avid binding than the dimeric IgG of the recombinant form. Eliminating IgM isotype antibodies by screening with isotype-specific secondary antibodies could avoid this problem.

### Recombinant antibodies have expected staining patterns on wholemount fixed tissue

**Figure 4:**
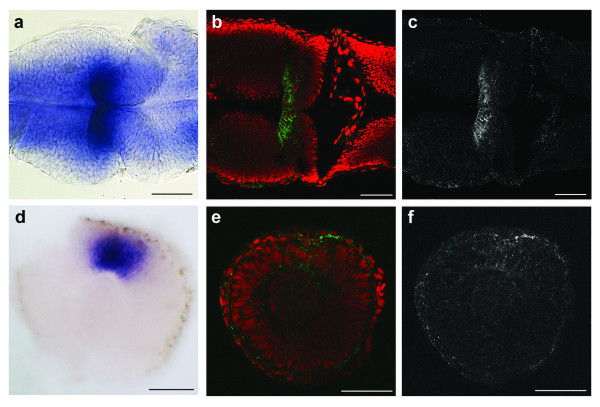
**Recombinant monoclonal antibodies recapitulate their gene expression patterns on wholemount fixed zebrafish embryos**. (a-c) Dorsal views of a 24 hpf zebrafish embryo showing *flrt3 *gene expression by *in situ *hybridization on the midbrain side of the mid-hindbrain boundary (a) and antibody staining with SI3-Flrt3 [green in (b), white in (c)]. (d-f) Lateral view of a dissected 24 hpf eye showing expression of the *unc5b *gene in the dorsal retina (d) and antibody staining with SI2-Unc5b [green in (e), white in (f)]. (b, e) red = DAPI nuclear stain. Scale bars = 50 μm.

In order to test our recombinant antibodies on wholemount fixed tissue, we determined the embryonic expression patterns of the *flrt3*, *unc5b *and *jamc.2 *genes and compared them to the antibody staining. At 24 hpf, *flrt3 *was expressed in a broad stripe at the mid-hindbrain boundary (Figure 4a) and the recombinant anti-Flrt3 antibody (SI3-Flrt3) also showed cell surface localization of the Flrt3 receptor in the same territory (Figure 4b, c). Similarly, *unc5b *was expressed in the dorsal part of the retina (Figure 4d) and this staining was recapitulated using the recombinant anti-Unc5b (SI2-Unc5b) antibody which showed cell surface staining in the dorsal retina (Figure 4e, f). Neither the *jamc.2 *gene nor SI4-Jamc.2 antibody showed staining on zebrafish embryos at different stages up to 48 hpf demonstrating that this gene was not expressed during early embryogenesis.

## Discussion

High quality, validated monoclonal antibodies are valuable biomedical reagents but their isolation can be time-consuming, unpredictable and expensive. By immunizing with pools of antigens and quickly cloning the productively rearranged variable antibody regions, we have greatly reduced the time, number of animals, and resources needed to isolate monoclonal antibodies that work on fixed wholemount tissues. Importantly, this procedure is accessible to smaller laboratories since it does not require expensive automated infrastructures or large clone libraries.

Antibodies that work in immunohistochemistry protocols are of most use for developmental and cell biologists but the protocols used often have long wash steps and therefore require high affinity antibodies. Here we have shown that the recombinant antibodies produced can have half-lives of many hours and are suitable for wholemount immunohistochemistry protocols. We have also shown that amino acid changes introduced within the variable regions by using degenerate primers to amplify the rearranged heavy and light fragments do not significantly affect the specificity or affinity of the recombinant antibody. In contrast, we observed a large decrease in binding avidity when the antibody isotype was changed from an avid decameric IgM to a bivalent IgG_1_. In order to avoid this problem, positive antibodies of the IgM isotype should be identified and eliminated at an early stage of the screening process using isotype-specific secondary antibodies.

Previous attempts to raise mouse monoclonal antibodies to zebrafish cell surface blood cell differentiation markers using fluorescence-activated cell sorting (FACS)-sorted zebrafish cells met with little success, most likely due to the presence of highly immunogenic glycans that were not protein-specific [20]. Zebrafish proteins expressed in a human cell line contain glycans that are not immunogenic in mice, making our recombinant protein library an ideal source of antigens for generating a zebrafish antibody resource. Importantly, although we have used this procedure to raise antibodies against cell surface zebrafish proteins, it could, in principle, be used to raise antibodies against any antigen that develops an immune response in mice, including intracellular proteins.

Recent technical advances could allow further refinements of the method: specificity screening on protein microarrays should permit to increase the number of pooled antigens (possibly 50 or more) used for the immunizations. Quicker immunization procedures such as the Repetitive Immunization at Multiple Sites (RIMMS) [30] in which antigens are administered over just an 11-day period could also further reduce the time taken to select antibodies. Methods for selecting monoclonal antibodies from single antibody-secreting cells have been recently developed but, crucially, do not enable collection of sufficient reagent for specificity screening before single cell RT-PCR amplification [31,32]. However, we have recently established that cell culture time can be reduced further by bypassing the 5-day expansion step before RT-PCR.

Cloning the productively rearranged light and heavy chains of selected monoclonal antibodies into a single mammalian expression vector facilitates their distribution and is an ideal format to build large reagent resources. It is also a very flexible system since additional properties such as purification tags (FLAG, poly-His) and reporter proteins (alkaline phosphatase, horseradish peroxidase or fluorescent proteins) can be easily added at the C-terminus of the heavy chain. Recent improvements in mammalian expression systems can now rapidly yield over 1 g/L of recombinant antibody from a transient transfection [33], raising new hopes for the rapid and large scale production of these valuable reagents.

## Conclusions

We have reported here the development of a procedure for cell and developmental biologists which streamlines the production of monoclonal antibodies recognizing antigens in wholemount fixed tissue. In this paper, we have used the method to raise antibodies against zebrafish proteins but it could also be broadly applied to any antigen that can raise a humoral immune response in mice. As only minimal equipment is required, this technique can also be used by smaller laboratories. Cloning of the rearranged light and heavy chains of selected monoclonal antibodies into a single mammalian expression vector facilitates their distribution, making scope for the creation of valuable antibody resources.

## Abbreviations

BSA: bovine serum albumin; CMV: cytomegalovirus; IgG: immunoglobulin G; PBS: phosphate buffered saline; PBST: PBS Tween; PCR: polymerase chain reaction; RT-PCR: reverse transcription PCR.

## Authors' contributions

CC designed the study and performed all the experiments except the wholemount immunohistochemistry, which was performed by NS, and the BIAcore studies, which were done by GJW. GJW conceived the study and wrote the manuscript which was edited by CC and NS. All authors read and approved the final manuscript.
